# Demographic and Psychosocial Factors Associated With Child Sexual Exploitation: A Systematic Review and Meta-analysis

**DOI:** 10.1001/jamanetworkopen.2020.17682

**Published:** 2020-09-22

**Authors:** Jessica J. Laird, Bianca Klettke, Kate Hall, Elizabeth Clancy, David Hallford

**Affiliations:** 1 School of Psychology, Faculty of Health, Deakin University, Burwood, Australia; 2 Center for Social and Early Emotional Development, Deakin University, Burwood, Australia; 3 Addictive and Anti-Social Behaviour Research, Deakin University Centre for Drug Use, Geelong, Australia

## Abstract

**Importance:**

Although research has examined factors associated with child sexual exploitation (CSE), consensus is lacking in regard to which factors should be prioritized, thereby hindering policy reform, prevention efforts, and development of early detection and intervention.

**Objective:**

To provide a meta-analytic synthesis of studies examining factors associated with CSE and to quantify their relative importance.

**Data Sources:**

Electronic databases searched to June 2019 included Medline, PsycINFO, the Cumulative Index to Nursing and Allied Health Literature, EMBASE, and Informit, yielding 396 nonduplicative records. Literature search was performed in July 2019.

**Study Selection:**

Inclusion criteria were quantitative investigations of sexual exploitation and mean sample age of 18 years or younger.

**Data Extraction and Synthesis:**

Literature review and data extraction followed Preferred Reporting Items for Systematic Reviews and Meta-analyses (PRISMA) guidelines. Thirty-seven studies met final inclusion criteria. Two independent reviewers extracted all relevant data. Random-effects meta-analyses were used to derive odds ratios (ORs) for each factor. Data were analyzed from September 1 to October 28, 2019, and prediction intervals calculated in June 2020.

**Main Outcomes and Measures:**

Child sexual exploitation, defined as coerced sexual acts between a child or a young person (aged ≤18 years) and an individual or a group in exchange for money, gifts, substances, or other commodities and associated factors.

**Results:**

Thirty-seven unique studies were included with a total of 67 453 unique participants (mean [SD] age of 16.2 [2.5] years; 49.9% female). Fifty-two factors associated with CSE were included in the meta-analysis. The strongest factors significantly associated with exposure to sexual exploitation were engagement in sexual risk behaviors (OR, 6.31 [95% CI, 3.12-12.76]; *P* < .001), having more than 5 sexual partners (OR, 5.96 [95% CI, 1.63-21.87]; *P* = .007), a diagnosis of posttraumatic stress disorder (OR, 5.29 [95% CI, 3.40-8.22]; *P* < .001), historical exposure to child pornography (OR, 5.50 [95% CI, 0.99-30.53]; *P* = .049), and a history of childhood sexual abuse (OR, 3.80 [95% CI, 3.19-4.52]; *P* < .001). A number of other potentially modifiable factors had moderate to strong associations.

**Conclusions and Relevance:**

In this systematic review and meta-analysis, children and adolescents affected by sexual exploitation showed high levels of sexual risk taking, multiple sexual partners, posttraumatic stress disorder, exposure to child pornography, and childhood trauma. Accurate detection of CSE may prevent this type of sexual violence occurring to adolescents and/or provide opportunities for intervention and recovery. Therefore, prevention and intervention efforts will likely benefit from integrating these factors into screening, assessment, and treatment.

## Key Points

**Question:**

What risk factors are associated with sexual exploitation in children?

**Findings:**

A systematic review and meta-analysis of 37 unique studies with 67 453 unique participants found 52 factors were associated with child sexual exploitation and available for meta-analysis. Results showed significant factors associated with exposure to sexual exploitation for children and youth are engagement in sexual risk behaviors, increased number of sex partners, posttraumatic stress disorder, exposure to child pornography, and a history of childhood sexual abuse.

**Meaning:**

Findings of this study suggest sexual risk behaviors, trauma, and exposure to sexual violence are key factors associated with sexual exploitation in children; results should inform future policy reform and prevention and intervention efforts.

## Teaser

This systematic review and meta-analysis provide a synthesis of studies examining factors associated with child sexual exploitation and quantify their relative importance.

## Introduction

Sexual exploitation is the second most lucrative crime in the world,^1,2,3,4^ estimated to affect up to 5% of the general child and youth population worldwide,^5,6^ with increasing numbers detected globally during the past decade.^7,8,9^ Although no unified global definition of child sexual exploitation (CSE) exists, it is considered a subtype of human trafficking.^1^ Definitions commonly include the actual or attempted abuse of a position of vulnerability, differential power, or trust over adolescents and children for sexual activity (online and/or offline) in exchange for something of value (eg, gifts, money, substances, or developmental needs, including shelter, food, and protection).^1,2^ Power imbalance between a perpetrator and young person is often characterized by age differences; however, age cutoffs vary greatly, making CSE difficult to clearly define and identify.^10,11^ Furthermore, CSE can occur between perpetrators and those exposed to sexual exploitation of the same age, and samples of young people can include emerging adults. Therefore, sexual exploitation vulnerability factors are likely more clinically relevant than singular age cutoffs in detecting and intervening for individuals affected by CSE. Nevertheless, to classify studies unequivocally, the present study focuses on CSE occurring in people 18 years or younger.

Research has indicated that sexually exploited young people are often psychologically controlled and manipulated by perpetrators; experience forcible isolation, rape, and extreme physical violence^12^; incur sexually transmitted infections^13,14^; and experience psychopathology, suicidality,^15,16,17^ and substance addiction.^18,19^ These outcomes have substantial economic costs, including service provision by child protection and health departments and long-term effects across the lifetime on the health and well-being of the individual.^20,21^

Previous research has identified numerous vulnerability factors associated with CSE, including psychological distress,^17^ emotion dysregulation,^22,23,24^ psychiatric symptoms,^15,16,17^ childhood trauma,^5,25^ poverty,^13,26^ single-parent families,^19,27,28^ criminality,^29,30,31^ and age.^19,32^ However, although these studies make an important contribution through the identification of discrete factors, no systematic synthesis of findings has been conducted to identify and quantify which factors are most critical and should be prioritized in CSE screening and intervention programs,^33^ for example, to investigate whether running away is associated with risk for CSE,^34,35^ a protective factor,^26^ or even unrelated.^11^

Furthermore, policy analysts, researchers, and professionals have advocated for multidimensional CSE prevention and intervention programs, which necessitate collaboration across health care providers, schools, and social services to support the needs of the child.^36^ However, despite growing documentation of CSE factors, the present evidence base only identifies unilateral risk factors or consists of studies insufficient in quality to meaningfully guide prevention and intervention.^33^

Therefore, to contribute to the elimination of all forms of trafficking and sexual exploitation, as outlined in the 2030 Agenda for Sustainable Development adopted by the United Nations,^37^ and to inform the development of effective CSE prevention and intervention, this meta-analysis aimed to synthesize the current evidence base of risk and protective factors associated with CSE and their estimated effect sizes. To our knowledge, this is the first meta-analytic review to examine and quantify factors associated with CSE.

## Methods

The review protocol was preregistered on PROSPERO (CRD42018100344). We followed the standards set by the Meta-analysis of Observational Studies in Epidemiology (MOOSE)^38^ and Preferred Reporting Items for Systematic Reviews and Meta-analyses (PRISMA) reporting guidelines.^39^

Included studies met the following inclusion criteria: (1) investigated sexual exploitation; (2) examined factors associated with sexual exploitation; (3) included children and young people (with a mean age of ≤18 years); (4) reported quantitative data; and (5) available in the English language. Studies were excluded if they (1) targeted adults (defined as mean age of sample >18 years); (2) described data qualitatively only; or (3) investigated sexual exploitation offenses.

Key terms (see eTable 1 in the Supplement) were searched via electronic databases in July 2019. The search included Medline, PsycINFO, the Cumulative Index to Nursing and Allied Health Literature, EMBASE, and Informit to June 2019. Articles were limited to peer-reviewed content. In addition, references of all included studies and gray literature (eg, government reports and working papers) were hand searched. Two authors (J.J.L. and B.K.) independently screened all titles and abstracts to determine which would proceed to full-text review. When reviewers were uncertain of a study’s eligibility, the full report was obtained, and discrepancies were discussed to obtain consensus.

Data collected from eligible studies included variables associated with CSE. Sexual exploitation definitions, sample size, study design, and study location were also extracted. If studies reported multiple effect sizes for the same variable, the effects were collapsed to avoid violating the independence of study effects. For example, variables such as living in foster care and living in residential care were collapsed as child protection involvement (see eTable 2 in the Supplement for full variable extraction information). When the same study sample was present across multiple publications, the largest sample size and the most comprehensive data extraction information was used. Studies were double coded, and discrepancies were resolved via consensus to maximize reliability and accuracy.

To examine the quality of methods and findings from included studies, 2 independent authors (J.J.L. and E.C.) evaluated each article, scoring them separately using a 9-point critical appraisal assessment tool adapted from Madigan et al^40^ (2018), based on previous meta-analytic research.^1,2,3,4,5,6,7,8,9,10,11,12,13,14,15,16,17,18,19,20,21,22,23,24,25,26,27,28,29,30,31,32,33,34,35,36,37,38,39,40,41,42,43,44^ Articles were given a score of 0 (no) or 1 (yes) for each criterion and summed to provide a total score of a possible 9. Higher scores correspond with higher methodological quality and lower risk of bias. Studies were categorized as low (<2), moderate (3-5), or high (≥6) quality. Full-quality assessment coding criteria and results are provided in eTables 2 and 3 in the Supplement.

### Statistical Analysis

Data were analyzed from September 1 to October 28, 2019. Prediction intervals were calculated in June 2020. Data were extracted and analyzed in Comprehensive Meta-Analysis software, version 3.^45^ A series of meta-analyses were conducted for each sexual exploitation factor, presented as an odds ratio (OR) with associated 95% CIs around the estimate. Effect sizes were weighted by the inverse of their variance, giving greater weight to studies with larger sample sizes, and thus more precision around estimates. Random-effects models were selected to calculate effect sizes, because they assume a distribution of effects across studies.

The *Q* and *I*^2^ statistics were computed to assess for statistical heterogeneity of effect sizes.^46,47^ A significant *Q* statistic suggests that study variability in effect size estimates is greater than the sampling error. The *I*^2^ statistic (ranging from 0%-100%) indicates the proportion of variability across studies owing to heterogeneity rather than chance. An *I*^2^ statistic of greater than 50% is indicative of at least moderate amounts of heterogeneity, although the statistic should be interpreted cautiously where there are few studies. Owing to variability of effects across different settings, prediction intervals are reported to evaluate between-study heterogeneity. A 95% prediction interval estimates where the true effects are expected for 95% of similar studies that might be conducted in the future.^48,49^ Two-tailed *P* < .05 indicated significance.

## Results

### Selected Studies

As shown in the PRISMA flow diagram (Figure), the electronic search yielded 396 nonduplicate records. A total of 112 articles were identified as potentially meeting inclusion criteria, with 37 full-text articles reviewed and included within the meta-analysis.

**Figure.**
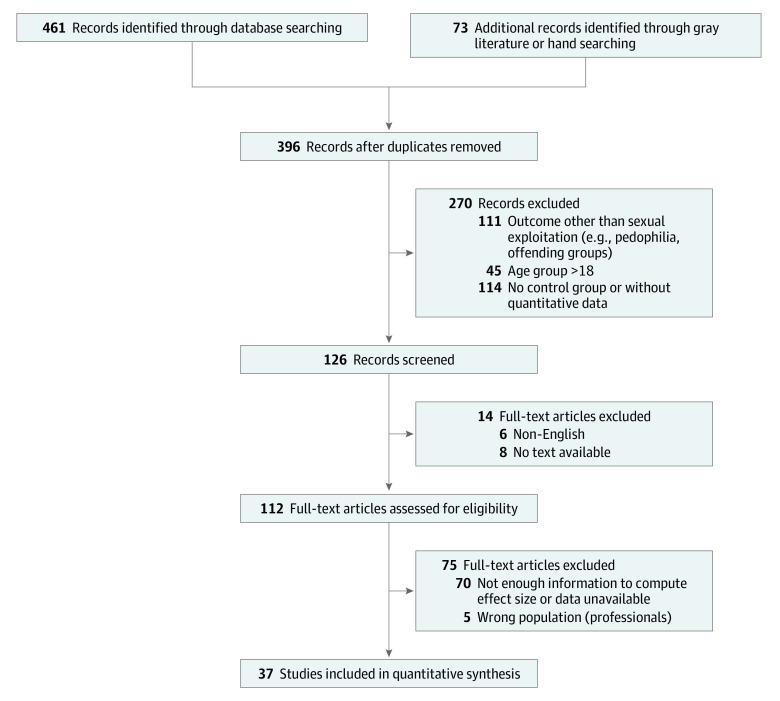
PRISMA Flow Diagram of Studies Included in the Systematic Review and Meta-analysis

### Study Characteristics

In total, 67 453 participants were included, with a mean (SD) age of 16.2 (2.5) years and near even distribution across sex (49.9% female and 50.1% male). The sample included 8.3% participants younger than 13 years; 2.3% aged 14 years, 34.3% aged 15 years, 44.8% aged 16 years, and 10.3% aged 17 to 18 years. Most studies were from the United States of America (n = 20), followed by Africa (n = 5), Sweden (n = 3), the United Kingdom (n = 2), and Canada (n = 3), with 1 study each from Taiwan, India, Norway, and the Philippines. All 37 articles^5,6,14,15,16,17,18,19,22,23,24,25,26,28,29,30,31,32,34,35,38,50,51,52,53,54,55,56,57,59,60,61,62,63,64,65,66^ were categorized in the high-quality range, with a mean (SD) study quality score of 8.4 (0.7) (see Table 1 and eTables 2 and 3 in the Supplement).

**Table 1.** Characteristics of the 37 Studies Included in the Meta-analysis by Study Design

Study design	Source	No. of participants	Age, mean (SD) [range], y	Female, No. (%)	Factors	Country	QAS
Cross-sectional	Adjei and Saewyc,^26^ 2017	1677	NR (NR) [12-17]^a^	838 (50.0)	Sex, alcohol use, interpersonal difficulties, physical abuse, poverty, school difficulties, sexual abuse, social isolation	Africa	9
	Atwood et al,^50^ 2012	714	16.4 (1.8) [14-17]	292 (40.9)	Age, age at first sexual experience	Africa	8
	Chohaney,^31^ 2016	328	NR (NR) [12-17]^b^	232 (70.7)	CALD, FSW, sex, homelessness, interpersonal difficulties, running away, school difficulties, YJI, emotional dysregulation, externalizing problems	United States	8
	Fredlund et al,^51^ 2018	5839	18.03 (NR) [12-18]	2919 (50.0)	Alcohol use, anxiety, depression, drug use, emotional dysregulation, hopeless/suicidal, psychological distress, PTSD, sexual abuse, SRB	Sweden	8
	Greenbaum et al,^52^ 2018	810	14.60 (NR) [11-17]	680 (84.0)	IPV, MSP, physical abuse, sexual abuse, sexual intercourse, SRB, STI/HIV, YJI	United States	8
	Ireland et al,^53^ 2015	198	20.18 (2.37) [NR]^c^^,^^d^	144 (72.7)	Anxiety, emotional dysregulation, sex, interpersonal difficulties, locus of control, self-esteem, social isolation	United Kingdom	8
	Lavoie et al,^24^ 2010	815	15.86 (0.74) [15-18]	464 (57.0)	Emotional dysregulation, experienced sex work, sex, household antisocial behavior, PornAdult, protective relationships, psychological distress, sexual abuse, sexual intercourse, SRB, substance use	Canada	9
	Layne et al,^54^ 2014	3785	15.30 (1.43) [13-18]	2346 (62.0)	Age, CALD, sex, repeated abuse	United States	8
	Lung et al,^55^ 2004	158	15.51 (1.50) [12-18]	158 (100)	Age, alcohol use, drug use, protective relationships, personality, single-parent family	Taiwan	7
	Martin et al,^56^ 2010	63	15.00 (NR) [15-17]^e^	63 (100)	Age at first sexual experience, age at first substance use, poverty, running away, SRB, STI/HIV, young parenthood	United States	7
	Naramore et al,^28^ 2017	102	16.30 (1.2) [12-18]	87 (85.3)	Sex, age, emotional abuse, family violence, antisocial household behavior, HMI, neglect, physical abuse, sexual abuse, single parent	United States	9
	O’Brien et al,^29^ 2017	800	16.70 (NR) [12-20]	0	Age, sexual abuse, SRB, YJI	United States	9
	O’Brien et al,^57^ 2017	814	13.65 (NR) [10-17]	488 (60.0)	CPI, drug use, emotional dysregulation, externalizing behavior, psychological distress, PTSD, running away	United States	8
	Panlilio et al,^6^ 2019	2400	12.74 (NR) [11-18]	1368 (57.0)	Age at first sexual experience, CPI, drug use, family violence, hopeless/suicidal, interpersonal difficulties, physical abuse, running away, school difficulties	United States	8
	Pedersen and Hegna,^58^ 2003	10 828	15.40 (0.9) [14-17]	5305 (49.0)	Age at first sexual experience, alcohol use, drug use, externalizing problems, MSP, physical abuse, social isolation, YJI	Norway	9
	Self-Brown et al,^59^ 2018	593	17.00 (NR) [NR]	332 (56.0)	Sex, age at first sexual experience, alcohol use, family violence, homelessness, antisocial household behavior, IPV, sexual abuse, SRB, social engagement	Africa	9
	Svedin and Priebe,^30^ 2007	60	15.90 (1.08) [14-18]	22 (36.7)	Age, CALD, sex, CPI, PornAdult, single parent, alcohol use, anxiety, depression, drug use, emotional dysregulation, interpersonal difficulties	Sweden	9
	Swahn et al,^60^ 2016	1134	15.04 (NR) [12-18]^f^	657 (57.9)	Age, alcohol use, CPI, family violence, sex, school difficulties, sexual abuse, single parent, STI/HIV	Africa	9
Retrospective	Chang et al,^14^ 2015	374	15.00 (NR) [13-17]	374 (100)	Age, CPI, externalizing problems, running away, sexual intercourse, SRB, STI/HIV	United States	7
	Fedina et al,^15^ 2019	273	14.30 (NR) [NR]	210 (76.9)	CALD, sex, CPI, emotional abuse, experienced sex work, homelessness, household antisocial behavior, psychological distress, running away, school difficulties, substance use, YJI	United States	8
	Fredlund et al,^22^ 2013	3498	15.40 (NR) [12-18]	1853 (53.0)	Protective relationships, interpersonal difficulties	Sweden	8
	Grosso et al,^61^ 2015	349	15.90 (NR) [NR]^g^	349 (100)	Sexual abuse, SRB, single parent, social engagement, STI/HIV	Africa	7
	Oram et al,^16^ 2015	96	14.23 (NR) [8-17]	65 (67.7)	CPI, depression, household antisocial behavior, physical abuse, psychological distress, PTSD, social isolation, substance use	United Kingdom	8
	Ulloa et al,^5^ 2016	11 620	16.18 (NR) [11-23]	6158 (53.0)	Age, age at first sexual experience, alcohol use, CALD, drug use, sex, homelessness, MSP, neglect, physical abuse, repeated abuse, running away, school difficulties, sexual abuse, sexual intercourse, STI/HIV, substance use	United States	9
	Wilson and Widom,^35^ 2010	1196	29.20 (3.8) [NR]^c^^,^^h^^,^^i^	586 (49.0)	Age at first sexual experience, age at first substance abuse, drug use, interpersonal difficulties, neglect, physical abuse, sexual abuse, running away, school difficulties, SRB, YJI	United States	9
	Yates et al,^66^ 1991	620	NR (NR) [12-24]^j^	403 (65.0)	Age at first sexual experience, alcohol use, CALD, depression, drug use, sex, homelessness, hopeless/suicidal, physical abuse, sexual abuse, psychological distress, school difficulties, STI/HIV	United States	8
Longitudinal	Edwards et al,^18^ 2006	13 294	16.20 (0.16) [12-18]	4387 (33.0)	Age, sex, alcohol use, cannabis use, depression, drug use, single parent, age at first sexual experience, sexual intercourse, STI/HIV	United States	9
	Kaestle,^19^ 2012	240	21.70 (0.11) [13-17]	120 (50.0)	Age, sex, CALD, cannabis use, depression, drug use, externalizing problems, homelessness, neglect, physical abuse, sexual abuse, protective relationships, single parent, social engagement, YJI	United States	9
	Reid,^25^ 2011	174	8.4 (3.34) [NR]^k^	174 (100)	Age at first sexual experience, emotional abuse, physical abuse, sexual abuse, neglect, poverty, running away, household antisocial behavior, HMI, SRB	United States	8
	Reid and Piquero,^32^ 2014	1354	16.04 (1.14) [13-18]	176 (13.0)	Age, age at first sexual experience, CALD, drug use, impulsivity, sex, household antisocial behavior, personality, repeated abuse, running away, sexual abuse, social engagement, young parenthood	United States	8
	Reid and Piquero,^62^ 2016	1354	16.04 (1.14) [13-18]	176 (13.0)	Age at first substance use, interpersonal difficulties, personality, emotional dysregulation, protective relationships, household antisocial behavior, psychological distress	United States	8
	Saewyc and Edinburgh,^17^ 2010	68	13.75 (1.13) [12-15]	68 (100)	Age, alcohol use, anxiety, CALD, drug use, emotional dysregulation, hopeless/suicidal, protective relationships, psychological distress, running away, self-esteem, sexting, sexual abuse, SRB, social engagement	Canada	8
	Salisbury et al,^34^ 2015	535	15.65 (NR) [9-19]^l^	144 (26.9)	Age, sex, CPI, homelessness, running away, YJI	United States	8
Observational	Deb et al,^23^ 2011	240	14.35 (NR) [13-18]	240 (100)	Emotional dysregulation, externalizing problems	India	7
	Reid,^63^ 2014	1714	8.4 (3.34) [NR]^h^	1714 (100)	Family violence, household antisocial behavior, IPV, sexual abuse, social engagement	United States	8
	Urada et al,^64^ 2014	770	NR (NR) [14-17]	770 (100)	Drug use, SRB, social engagement, STI/HIV, substance use, YPI	Philippines	9
Mixed methods	Nadon et al,^65^ 1998	82	16.30 (NR) [13-18]	82 (100)	Drug use, family violence, homelessness, physical abuse, running away	Canada	7

Abbreviations: CALD, culturally and linguistically diverse community; CPI, child protection involvement; FSW, family involved in sex work; HMI, household mental illness; hopeless/suicidal, hopelessness and suicidality; IPV, intimate partner violence; MSP, multiple sexual partners (>5); NR, not reported; PornAdult, exposure to adult pornography; PTSD, posttraumatic stress disorder; QAS, Quality Assessment Scoring; SRB, sexual risk behaviors; STI, sexually transmitted infection; YJI, youth justice involvement.

^a^ Sample size of 1314 males (983 aged 12-17 years; 331 aged 18-19 years) and 844 females (694 aged 12-17 years; 150 aged 18-19 years).

^b^ Median age of 35 years reported.

^c^ Analyzed the occurrence of sexual exploitation at younger than 18 years retrospectively via an adult sample.

^d^ Checklist to assess sexual exploitation examines experiences of participants when they were younger than 16 years.

^e^ Median age of first sex trade for juvenile starters (<18 years when commenced sexual exploitation) was 15 (range, 15-17) years. Mean age of respondents at the time of survey was 37 (range, 18-70) years.

^f^ Age was categorized into 3 categories: 12 to 14 years (n = 23), 15 to 16 years (n = 39), and 17 to 18 years (n = 20).

^g^ Indicates mean age of retrospective time of reporting at 2 locations (21.7 years in Ouagadougou; 25.0 years in Bobo-Dioulasso). Sexually exploited group categorized as started at younger than 18 years.

^h^ Participants were asked whether they had “exchanged sex for money or drugs, that is, engaged in prostitution up to and including the age of 17.”

^i^ Age range was 10 to 14 years (n = 21), 15 to 17 years (n = 206), and 18 to 21 years (n = 53). Most were younger than 18 years.

^j^ Participants were restricted to cases of children 11 years or younger at the time of the incident. Interviews to collect retrospective data occurred at a mean of 29 years of age. Sexual exploitation occurred at younger than 11 years.

^k^ Mean (SD) age at hospital visit in 1973 to 1975 was 8.4 (3.34) years; at interview in 1996 to 1997, 31.6 (3.30) years. Sexual exploitation was measured at younger than 18 years (yes or no).

^l^ Ninety-eight percent of the sample were aged 9 to 18 years; 0.9%, 19 years (n = 5).

### Factors Associated With CSE

A total of 52 factors associated with CSE were available for meta-analysis (see Table 2). These factors were collapsed across 6 domains, including demographic, trauma and exposure to abuse and/or violence, internalizing problems (ie, internally focused symptoms), externalizing problems (ie, externally focused behavioral symptoms), and psychosocial and protective domains.^67^

**Table 2.** Factors Associated With CSE

Characteristic	Main effects			Heterogeneity			
	No. of studies	OR (95% CI)	*P* value	*Q* statistic	*P* value	*I*^2^, %	95% PI
Demographic							
Age	13	0.49 (0.35-0.69)	<.001	806.42	<.001	98	0.12-1.99
Female	16	2.25 (1.52-3.32)	<.001	327.25	<.001	95	0.46-10.89
CALD	10	2.57 (1.95-3.39)	<.001	43.17	<.001	79	1.04-6.35
Trauma and exposure to abuse and/or violence							
Exposure to child pornography	3	5.50 (0.99-30.53)	.049	27.99	<.001	92	0.009^a^
PTSD	3	5.29 (3.40-8.22)	<.001	1.75	.42	0	3.40-8.22
Sexual abuse	19	3.80 (3.19-4.52)	<.001	37.26	.005	51	2.27-6.35
Exposure to violent/rape pornography	3	2.76 (1.08-7.04)	.03	12.10	.002	83	0.10-76.30
Intimate partner violence	3	2.57 (1.47-4.47)	.001	6.66	.03	67	0.41-16.22
Neglect	5	2.15 (1.52-3.03)	<.001	8.57	.07	53	0.92-4.99
Physical abuse	14	1.61 (1.34-1.92)	<.001	34.85	<.001	62	0.95-2.71
Emotional abuse	3	1.60 (1.04-2.46)	.003	0.47	.79	0	1.04-2.46
Repeated exposure to abuse and/or violence	4	1.35 (1.12-1.64)	.002	9.37	.03	67	0.81-2.25
Family violence	7	1.22 (0.83-1.79)	.32	22.49	.001	73	0.42-3.49
Externalizing problems							
Sexual risk behaviors^b^	14	6.31 (3.12-12.76)	<.001	340.23	<.001	96	0.39^c^
Mean lifetime No. of sex partners	5	5.96 (1.63-21.87)	.007	159.98	<.001	97	0.09^d^
Externalizing problems	10	3.50 (1.98-6.07)	<.001	70.49	<.001	87	0.61-19.81
Sexting (ever sent)	2	3.12 (1.92-5.10)	<.001	1.74	.19	42	0.15-63.95
Criminality	11	3.10 (1.50-2.16)	<.001	52.01	<.001	80	0.69-6.71
Sexual intercourse (ever had)	8	2.98 (1.99-4.45)	<.001	26.70	<.001	73	0.93-9.55
Drug use	17	2.89 (1.73-3.03)	<.001	187.21	<.001	91	1.03-8.15
Interpersonal difficulties	6	2.29 (1.54-3.41)	<.001	41.95	<.001	88	0.72-7.25
Running away (ever)	15	2.28 (1.63-3.19)	<.001	165.79	<.001	91	0.60-8.61
AOD overall	16	2.16 (1.67-2.79)	<.001	169.37	<.001	91	0.85-5.49
School difficulties	11	2.15 (1.57-2.94)	<.001	107.22	<.001	90	0.69-6.64
Marijuana use	5	1.85 (1.42-2.42)	<.001	9.01	.06	55	0.98-3.52
Alcohol use	15	1.69 (1.42-2.02)	<.001	64.93	<.001	78	0.93-3.06
Age at first sexual experience	12	1.35 (1.10-1.69)	.01	192.76	<.001	94	0.59-3.08
Age of initial substance use	4	1.30 (0.74-2.29)	.36	50.53	<.001	94	0.21-8.20
Interpersonal difficulties with caregivers	6	1.25 (0.88-1.79)	.21	65.56	<.001	92	0.72-7.26
Internalizing problems							
Anxiety	5	3.11 (2.13-4.50)	<.001	12.47	.01	68	1.15-8.42
Emotion dysregulation	10	2.91 (1.86-2.33)	<.001	38.44	<.001	76	1.25-4.36
Psychological distress	10	2.76 (1.86-4.01)	<.001	24.54	.003	63	0.94-8.09
Hopelessness and suicidality	6	2.64 (1.48-4.71)	.001	49.61	<.001	89	0.45-15.33
Locus of control	3	2.15 (1.36-3.42)	.001	2.67	.26	24	0.78-5.94
Depression	7	2.10 (1.27-3.46)	.004	37.34	<.001	83	0.47-9.42
Psychoticism, personality trait	2	1.09 (0.46-2.62)	.84	6.96	.008	85	0.001^e^
Self-esteem	2	0.80 (0.19-3.42)	.76	20.05	<.001	95	0.000^f^
Psychosocial							
STI/HIV (ever had)	10	2.90 (1.50-5.71)	.002	83.24	<.001	89	0.29-28.75
Single-parent family	7	2.75 (1.48-5.11)	.001	49.74	<.001	87	0.40-18.63
Homelessness	8	2.22 (1.75-2.81)	<.001	12.78	.08	45	1.25-3.93
Family involved in sex work	3	1.84 (1.11-3.04)	.02	0.47	.79	0	1.11-3.04
Poverty	6	1.80 (1.19-2.72)	.005	19.61	<.001	74	0.61-5.38
Child protection involvement	8	1.64 (1.14-2.35)	.008	76.13	<.001	90	0.59-4.54
Social isolation	6	1.62 (1.19-2.20)	.002	12.54	.03	60	0.76-3.46
Household antisocial behaviors	8	1.52 (1.19-1.94)	.001	9.06	.25	22	0.95-2.42
Stressful life events	2	1.15 (0.59-2.24)	.69	4.33	.04	76	0.005^g^
Exposure to pornography (heterosexual)	2	0.72 (0.40-1.30)	.28	1.19	.28	15	0.08-6.80
Young parenthood (self) (<18 y)	3	0.70 (0.16-3.01)	.63	16.12	<.001	87	0.003^h^
Household mental illness	2	0.65 (0.11-4.00)	.06	5.13	.02	80	0.000^i^
Protective							
Protective relationships (extrafamilial)	3	1.14 (0.49-2.63)	.76	158.34	<.001	98	0.05-26.57
Social engagement (school or work)	5	0.83 (0.65-1.05)	.12	13.35	<.001	70	0.44-1.57
Protective relationships (intrafamilial)	4	0.68 (0.41-1.15)	.16	24.98	<.001	88	0.14-3.50

Abbreviations: AOD, alcohol and other drug use; CALD, culturally and linguistically diverse community; CSE, child sexual exploitation; OR, odds ratio; PI, prediction intervals; PTSD, posttraumatic stress disorder; STI, sexually transmitted infection.

^a^ Upper 95% prediction interval is 3243.65.

^b^ Include condomless sex, sexual intercourse in public, and meeting with strangers face-to-face from an online environment for sex.

^c^ Upper 95% prediction interval is 101.89.

^d^ Upper 95% prediction interval is 400.13.

^e^ Upper 95% prediction interval is 1919.97.

^f^ Upper 95% prediction interval is 391 013.24.

^g^ Upper 95% prediction interval is 265.99.

^h^ Upper 95% prediction interval is 149.08.

^i^ Upper 95% prediction interval is 2 739 269.2.

Thirteen studies^5,14,17,18,19,28,30,32,50,54,55,57,60^ were available to estimate the pooled effect size for the association between age and CSE (see Table 2), with mean participant ages ranging from 8 to 17 years. A random-effects analysis produced a significant combined effect size (OR) of 0.49 (95% CI, 0.35-0.69), indicating that for every year of age, the odds of being sexually exploited were 50% less (eg, an individual aged 16 years was half as likely to be sexually exploited compared with one aged 15 years). Female participants were twice as likely to experience CSE compared with male participants (OR, 2.25 [95% CI, 1.52-3.32]). Young people from culturally and linguistically diverse communities were more than twice as likely to experience CSE when compared with those who identified as White (OR, 2.57 [95% CI, 1.95-3.39]). Heterogeneity was high for age and sex and moderate for culturally and linguistically diverse variables (see Table 2).

Of the 10 factors within this domain, 9 adverse childhood experiences (potentially traumatic events that occur from 0 to 17 years of age) were associated with increased odds of experiencing CSE in adolescence (see Table 2). A history of childhood sexual abuse increased the odds by nearly 4 times (OR, 3.80 [95% CI, 3.19-4.52]). Neglect (OR, 2.15 [95% CI, 1.52-3.03]) and physical (OR, 1.61 [95% CI, 1.34-1.92]) and emotional (OR, 1.60 [95% CI, 1.04-2.46]) abuse doubled the odds. Numerous episodes of physical or sexual abuse before CSE increased the odds of exposure to CSE by 1.35 (95% CI, 1.12-1.64]). Evidence suggested that exposure to child pornography (OR, 5.50 [95% CI, 0.99-30.53]) or a history of posttraumatic stress disorder (PTSD) (OR, 5.29 [95% CI, 3.40-8.22]) were associated with experience of CSE in adolescents, increasing risk 5-fold. Exposure to violent or rape pornography (OR, 2.76 [95% CI, 1.08-7.04]), intimate partner violence (OR, 2.57 [95% CI, 1.47-4.47]), or a family member involved in sex work (OR, 1.84 [95% CI, 1.11-3.04]) increased the odds of experiencing CSE from 2 to 5 times. Family violence was not found to be associated with CSE (OR, 1.22 [95% CI, 0.83-1.79]). Heterogeneity was high for exposure to child or violent pornography and moderate for intimate partner violence, neglect, sexual/physical abuse, repeated episodes of violence and/or abuse, and family violence. No heterogeneity was reported across PTSD or emotional abuse.

The strongest externalizing factors associated with CSE (Table 2) were sexual risk behaviors (ie, condomless sex, sex in public, or meeting strangers from an online chat for physical sex) (OR, 6.31 [95% CI, 3.12-12.76]) and reporting multiple sexual partners in a lifetime (OR, 5.96 [95% CI, 1.63-21.87]), increasing risk 6-fold. Externalizing problems, such as aggression and hostility toward others (OR, 3.50 [95% CI, 1.98-6.07]) and a lifetime history of committing a crime (OR, 3.10 [95% CI, 1.50-2.16]), sending a sext message (sexually explicit content via electronic device) (OR, 3.12 [95% CI, 1.92-5.10]), and being sexually active (OR, 2.98 [95% CI, 1.99-4.45]), were found to triple the odds of experiencing CSE. Alcohol and drug use (OR, 2.16 [95% CI, 1.67-2.79]), running away (OR, 2.28 [95% CI, 1.63-3.19]), interpersonal difficulties (OR, 2.29 [95% CI, 1.54-3.41]), or conflict at school (OR, 2.15 [95% CI, 1.57-2.94]) doubled the likelihood of experiencing CSE. Age of first sexual experience (OR, 1.35 [95% CI, 1.10-1.69]) and age at initial substance use (OR, 1.30 [95% CI, 0.74-2.29]) increased the odds of CSE by nearly 1.5 times. Interpersonal difficulties with caregivers was not statistically significant (OR, 1.25 [95% CI, 0.88-1.79]). Heterogeneity was moderate for sexting and marijuana use and high for all other externalizing factors.

Factors associated with CSE within the internalizing problems domain included anxiety symptoms (OR, 3.11 [95% CI, 2.13-4.50]), emotional dysregulation (OR, 2.91 [95% CI, 1.86-2.33]), psychological distress (OR, 2.76 [95% CI, 1.86-4.01]), and hopelessness and suicidality (OR, 2.64 [95% CI, 1.48-4.71]), all of which nearly tripled the odds of experiencing CSE (see Table 2). Depression (OR, 2.10 [95% CI, 1.27-3.46]), social isolation (OR, 1.62 [95% CI, 1.19-2.20]), and locus of control (OR, 2.15 [95% CI, 1.36-3.42]) doubled the odds of experiencing CSE. Self-esteem issues (OR, 0.80 [95% CI, 0.19-3.42]) and psychoticism (OR, 1.09 [95% CI, 0.46-2.62]) were not significant. Heterogeneity was moderate for locus of control and anxiety and high for remaining internalizing factors.

Psychosocial factors significantly associated with sexual exploitation include a history of having a sexually transmitted infection (OR, 2.90 [95% CI, 1.50-5.71]) or being part of a single-parent household (OR, 2.75 [95% CI, 1.48-5.11]), increasing the odds of experiencing CSE 3-fold. Adolescents who reported a history of homelessness (OR, 2.22 [95% CI, 1.75-2.81]), family income below the poverty line (OR, 1.80 [95% CI, 1.19-2.72]), or child protection involvement (OR, 1.64 [95% CI, 1.14-2.35]) had a 2-fold risk. Youths exposed to household antisocial behaviors (ie, criminality) were 1.5 times as likely to experience CSE (OR, 1.52 [95% CI, 1.19-1.94]). Stressful life events in general (OR, 1.15 [95% CI, 0.59-2.24]), household mental illness (OR, 0.65 [95% CI, 0.11-4.00]), exposure to heterosexual pornography (OR, 0.72 [95% CI, 0.40-1.30]), and early parenthood themselves (<18 years of age) (OR, 0.70 [95% CI, 0.16-3.01]) were not significantly associated with CSE exposure. Heterogeneity was little or none for family involved in sex work, household antisocial behaviors, and exposure to pornography; moderate for social isolation and homelessness; and high for the remaining psychosocial factors.

Social engagement, including school completion or employment (OR, 0.83 [95% CI, 0.65-1.05]) and protective relationships within the family (OR, 0.68 [95% CI, 0.41-1.15]) or outside of the family unit (OR, 1.14 [95% CI, 0.49-2.63]), was not significantly associated with CSE exposure.

### Factors Associated With CSE Based on Longitudinal Study Design

A total of 19 factors were extrapolated from longitudinal data (Table 3). Similar to the complete sample of studies, young people and children who experienced sexual exploitation were significantly more likely to experience child sexual abuse (OR, 2.89 95% CI, 1.70-4.58]), running away (OR, 2.75 [95% CI, 1.75-4.31]), homelessness (OR, 2.22 [95% CI, 1.68-2.92]), emotional dysregulation (OR, 1.69 [95% CI, 1.18-2.41]), alcohol use (OR, 1.54 [95% CI, 1.24-1.93]), and/or externalizing problems (OR, 1.37 [95% CI, 1.08-1.73]) when compared with nonexploited youth. In contrast, there were no significant differences as a function of sex, physical abuse, marijuana use, age at first sexual experience, or depression. Age at initial substance use and social engagement with school or work remained nonsignificant for associations with CSE; however, protective relationships within the family significantly reduced the likelihood of experiencing CSE in adolescence (OR, 0.84 [95% CI, 0.71-0.99]).

**Table 3.** Factors Associated With CSE by Longitudinal Study Design

Factor	Main effects			Heterogeneity			
	k	OR (95% CI)	*P* value	*Q* statistic	*P* value	I^2^, %	95% PI
Demographic							
CALD	4	3.12 (1.78-5.41)	<.001	21.69	<.001	86	0.24-39.35
Female	4	1.60 (0.74-3.47)	.23	53.09	<.001	94	0.04-58.39
Age	3	1.05 (0.96-1.14)	<.001	2.98	.23	33	0.47-2.32
Trauma and exposure to abuse and/or violence							
Sexual abuse	4	2.89 (1.70-4.58)	<.001	5.44	.14	45	0.86-8.95
Neglect	2	1.52 (1.01-2.28)	.04	1.08	.30	7	0.52-4.39
Physical abuse	2	1.34 (0.80-2.24)	.27	2.27	.13	56	0.03-49.33
Externalizing problems							
Running away (ever)	6	2.75 (1.75-4.31)	<.001	33.48	<.001	86	0.68-11.03
Drug use	4	1.99 (1.39-3.47)	.02	56.69	<.001	95	0.36-10.91
Alcohol use	3	1.54 (1.24-1.93)	<.001	4.84	.09	59	0.77-3.07
Externalizing problems	2	1.37 (1.08-1.73)	.009	3.28	.07	70	0.22-8.33
Marijuana use	3	2.74 (0.92-8.17)	.07	54.21	<.001	96	0.04^a^
Age at initial substance use	2	0.90 (0.27-2.97)	.86	16.42	<.001	94	0.000^b^
Age at first sexual experience	3	0.88 (0.72-1.08)	.20	9.78	.01	80	0.43-1.78
Internalizing problems							
Emotion dysregulation	3	1.69 (1.18-2.41)	.004	23.44	<.001	91	0.45-6.30
Depression	2	1.62 (0.78-3.35)	.19	7.09	.01	86	0.003^c^
Psychosocial							
Homelessness	2	2.22 (1.68-2.92)	<.001	.003	.96	0	2.21-3.84
Single-parent family	2	1.19 (0.94-1.50)	.15	.15	.69	0	0.94-1.50
Protective							
Protective relationships (intrafamilial)	2	0.84 (0.71-0.99)	.04	1.16	.28	13	0.46-1.52
Social engagement (school or work)	2	0.93 (0.70-1.24)	.62	2.16	.14	53	0.13-6.80

Abbreviations: CALD, culturally and linguistically diverse community; CSE, child sexual exploitation; OR, odds ratio; PI, prediction intervals.

^a^ Upper 95% prediction interval is 185.80.

^b^ Upper 95% prediction interval is 38 129.34.

^c^ Upper 95% prediction interval is 810.49.

## Discussion

Child sexual exploitation remains a major global problem demanding an evidence-based response. Although a plethora of vulnerability factors for CSE have been examined, the present study is the first, to our knowledge, to systematically synthesize and quantify factors associated with children and adolescents (≤18 years) affected by sexual exploitation and to ascertain which factors are the most significant. Findings highlighted young people who commonly experience recurring trauma and violence before exploitation and consequently experience psychological symptoms associated with prior exposure to abuse and/or violence and trauma as the strongest factors associated with CSE.

The most impactful risk factors associated with CSE are clustered around early, risky, and abusive sexual behaviors. These include sexual risk-taking behaviors (eg, condomless sex, sexual intercourse in public, or meeting face-to-face with strangers from an online environment for sex), multiple sex partners (>5), and exposure to child pornography, which increases the likelihood of exposure to CSE by as much as 6-fold. These findings are consistent with research that suggests exposure to sexually exploitative material and experiences at a young age contribute to ongoing risk behaviors and increased likelihood of exploitation in adolescence.^68,69^ Similarly, adolescents who experienced sexual abuse or have engaged in electronic sexting behaviors are 3 to 4 times as likely to experience CSE.^70^ Research consistently reports sexual risk taking, online and offline, to be linked with adverse mental health outcomes and future exploitation.^70,71^ Online sexual violence research also suggests that pornography and sexting behaviors can function as extensions of offline forms of sexual coercion,^72,73^ which may explain our findings associating several online sexual risk behaviors with the physical experience of CSE. Furthermore, exposure to or exchanging sexually explicit content online can act as a potential vehicle for online grooming and can be a form of CSE itself.^74^

Consequent to exposure to abuse and/or violence, it is not surprising that symptoms associated with trauma, including a diagnosis of PTSD, aggression or hostility toward others (ie, externalizing problems), anxiety, emotional dysregulation, and psychological distress were associated with a 3- to 5-fold increase in the likelihood of CSE. Neurobiological evidence indicates that unresolved trauma interferes with functioning in daily life and the capacity to regulate arousal, emotions, and behavior.^75,76^ For example, atypical regulatory systems develop to cope with threat when trauma is experienced at a young age,^75^ and although these strategies are effective in the short term (eg, running away or substance use), they are risky and damaging in the long term.^77^

A cluster of psychosocial vulnerabilities and demographic factors were also found to be associated with CSE, including age and sex. Specifically, results indicate younger age and female sex are implicated in CSE vulnerability, mirroring research that reports young women are twice as likely to experience sexual violence before 15 years of age when compared with young men.^78^ Some research reports other factors, such as PTSD, may be a greater determinant of future sexually violent situations independently of sex.^79^ The present study supports the notion that PTSD is a significantly stronger factor than sex, but these factors may still interact. Furthermore, results affirm research that indicates children and young people affected by HIV, poverty, homelessness, and broken homes are vulnerable to CSE at higher rates than their peers.^80^

Three protective factors were available for meta-analysis, and although protective relationships (extrafamilial and intrafamilial) and social engagement were not significantly associated with CSE in the complete sample of studies, separate analysis of these factors based on longitudinal design alone found close family relationships may protect young people from CSE. However, data were largely based on studies using samples from child protection and criminal justice services, thus limiting generalizability in regard to relational protective factors.

Seven studies^17,18,19,25,32,34,62^ based on a longitudinal design reported significant variation across effect sizes. Although findings were largely heterogenous, sex and internalizing factors, including psychological distress and depression, were not associated with CSE. These results are preliminary owing to limited data availability, and further investigation is warranted. However, longitudinal data provided further evidence that trauma and early childhood events of exposure to abuse and/or violence remain closely associated with future experiences of CSE. Although these events cannot be undone, trauma symptoms can be tempered by evidence-based interventions that assist with emotion regulation and trauma processing, supporting a young person’s pathway into recovery.^81,82^

A final finding of this study lies in confirmed variability among factor effect sizes between studies. Although seeking clarity regarding which factors are important for CSE intervention and prevention initially drove the conduct of this study, heterogenous findings further highlight the inconsistency and dearth of research within the area of CSE. Practice, policy, and, most importantly, the adolescents and children reflected in this research require further exploration to more wholly understand pathways into and out of sexual exploitation. Consequently, without further research, the development of efficacious prevention and intervention to eliminate this type of violence against children and youth may be hindered.

### Clinical Implications

Based on our findings, earlier identification of CSE factors through screening is paramount in preventing further sexual exploitation of vulnerable children and adolescents. Most tertiary and primary health settings frequently screen for other risks, such as substance use and suicidality; however, despite the pervasiveness of sexual violence and CSE, these are rarely a focus of screening.^79,80^ Youth presenting to health services with sexual risk behaviors, sexually transmitted infection and HIV testing, a history of sexual violence online or offline, and a profile of trauma symptoms (eg, PTSD, externalizing problems, anxiety, or emotional dysregulation) warrant thorough assessment for potential CSE risk.

Prior research implicates unresolved childhood trauma as the single most significant factor associated with subsequent contact with the mental health system.^83^ Although current CSE intervention research is limited, most programs focus on broad psychosocial issues, such as prevention of homelessness or sexually transmitted infection,^33^ and do not address the need for psychological treatment of trauma symptoms. Furthermore, meta-analytic research indicates that efficacious CSE intervention programs should include both knowledge and skill building; however, few studies include both.^33,82^ Finally, although psychoeducational programs with respectful relationships curricula can improve healthy relationship knowledge and attitudes for youth in schools,^84^ these programs are not tailored to meet the complex needs of intervention for youth affected by CSE, especially given the overrepresentation of disengagement from formal school settings in youth populations affected by trauma.^85^ Therefore, our results suggest future CSE interventions would benefit from integrating the psychological treatment of trauma symptoms (eg, PTSD, externalizing disorders, emotion dysregulation, and anxiety) with a psychoeducation program that includes sexual safety both online and offline.

### Limitations

The present results should be interpreted with the understanding that the findings are correlational and cannot imply causation of CSE. Further, there were discrepancies in the number of studies and sample sizes across factors, leading to some large variances in effect size estimates. Caution should also be exercised owing to the high proportion of data sampled from criminal records and child protection, which may bias generalizability. Further, some variables, such as self-esteem and personality, had very few studies included in the meta-analysis. Finally, because several identified factors are often co-occurring, additive and interactive effects are recommended for exploration in future research.

## Conclusions

Risk factors associated with CSE must be addressed to prevent this type of sexual violence and to provide pathways for recovery for affected young people. The present study reports findings consistent with research that suggests early experiences of sexual violence may distort interpersonal relationships,^86,87,88^ normalize sexual risk,^89^ strengthen stereotypes regarding sex and violence,^90^ and perpetuate ongoing repeated exposure to violence and/or abuse.^91^ According to our meta-analytic results, adolescents or children presenting to primary or tertiary services with risky sexual behaviors, prior exposure to sexual violence online and offline, and mental health risk factors associated with trauma warrant further assessment for CSE. Trauma-informed intervention planning and design for youth affected by CSE should incorporate the psychological treatment of trauma symptoms alongside supportive psychoeducation regarding sexual safety online and offline. This review informs the current evidence base and the design of initiatives seeking to prevent and intervene early for CSE among children and adolescents.^50^

## Notes

Supplement. **eTable 1.** Search Strategy **eTable 2.** Criteria for Assessing Study Quality for All Studies Included in the Meta-analysis **eTable 3.** Study Quality Scoring for Each Study Included in the Meta-analysis **eReferences**
