# Supplementary material for: Demographic and Psychosocial Factors Associated With Child Sexual Exploitation: A Systematic Review and Meta-analysis
Source: JAMA Netw Open. 2020 Sep 22;3(9):e2017682. doi: 10.1001/jamanetworkopen.2020.17682 (PMC7509625; doi:10.1001/jamanetworkopen.2020.17682)
Supplement: Supplement. — eTable 1. Search Strategy eTable 2. Criteria for Assessing Study Quality for All Studies Included in the Meta-analysis eTable 3. Study Quality Scoring for Each Study Included in the Meta-analysis eReferences [file jamanetwopen-e2017682-s001.pdf]

Supplementary Online Content

Laird JJ, Klettke B, Hall K, Clancy E, Hallford D. Demographic and psychosocial factors associated with child sexual exploitation: a systematic review and meta-analysis. *JAMA Netw Open*. 2020;3(9):e2017682. doi:10.1001/jamanetworkopen.2020.17682

eTable 1. Search Strategy

eTable 2. Criteria for Assessing Study Quality for All Studies Included in the Meta-analysis

eTable 3. Study Quality Scoring for Each Study Included in the Meta-analysis

eReferences

This supplementary material has been provided by the authors to give readers additional information about their work.

©2020 Laird JJ et al. *JAMA Network Open*.

eTable 1. Search Strategy

Search Number	Search Terms Included
1	“sexual exploitation” OR “sexual exploitation of children” OR "survival sex" OR "transaction* sex" OR "sex traffic*” OR “commercial sexual exploitation of children” OR “domestic minor sex traffic*” OR "commercial sexual exploitation" OR "child sexual exploitation” OR prostitution OR "sex work*" OR “trading sex” OR “selling sex” OR “child porn*” OR “Sex industry” OR “sex N4 swap” OR “Sex exchange*” OR “sex N4 exchange*”
2	"young people" OR youth* OR adolescen* OR "young person*" OR minor* OR teen* OR child* OR juvenile*
3	vulnerab* OR risk* OR "risk factor*" OR "protective factor*" OR coping OR protect* OR sociocultural* OR psychosocial* OR resilien* OR hardiness OR correlate* OR indicator* OR determin* OR predict* OR factor* OR characteristic* OR sequalae* OR strength*

Notes: Databases searched: MEDLINE, EMBASE, PsycINFO, CINAHL, Informit. **Limiters**: All in title, peer-reviewed.

©2020 Laird JJ et al. *JAMA Network Open*.

eTable 2. Criteria for Assessing Study Quality for All Studies Included in the Meta-analysis

Criterion	Description	Scoring
1. Defined Sample	Does the study have a defined sample based on the following elements?: • defined eligibility and exclusion criteria • age range/cut-offs • an adequate description of the recruitment process The study must meet at least 2 of the above elements to receive a score of 1.	Yes = 1; No = 0
2. Representative Sample	Is the study sample representative of the specific population that it draws from? If representativeness is unspecified, score as 0.	Yes = 1; No = 0
3. Controls in Analysis	Is the sample weighted or controlled for factors such as gender and age? Does the study include a regression analysis to take into account the effect of moderating variables?	Yes = 1; No = 0
4. Predictors Measured	Does the study measure and report findings on at least one predictor other than gender?	Yes = 1; No = 0
5. Completion Rate	Does the study report a completion rate?	Yes = 1; No = 0
6. Demographic Info	Does the study provide adequate demographic information? The study must report at least the sample age (mean age or arrayed in age bands) *and* 1 other element (e.g., gender, ethnicity).	Yes = 1; No = 0
7. Definition Provided	Is child sexual exploitation clearly defined?	Yes = 1; No = 0
8. Sexting Exploitation Factors	Does the study provide quantitative details (excluding gender and ethnicity) on factors associated with sexual exploitation? For example, are factors provided for sub-groups or specific factors associated with sexual exploitation? At least three details need to be reported to receive a score of 1. Examples include: • sexual exploitation factors measured through quantitative data analysis • sexual exploitation factors are provided for sub-groups (e.g., age) • sexual exploitation factors are quantitatively measured individually providing an effect size for a specific associated factor	Yes = 1; No = 0
9. Publication Status	Is the study published (peer-reviewed journals, book chapters)?	Yes = 1; No = 0

Note: Methodological quality of reported findings were assessed via a 9-point critical appraisal assessment tool developed by Madigan et al, 2018. Articles are given a score of 0 (no) or 1 (yes) for each criterion and summed to give a total score out of 9. The classification used identified studies of low (≤2), moderate (3-5) or high (≥6) quality.

Tool adapted from the following; Madigan S, Ly A, Rash CL, Van Ouytsel J, Temple JR. Prevalence of multiple forms of sexting behavior among youth: a systematic review and meta-analysis. *JAMA Pediatr*. Published online February 26, 2018. doi:10.1001/jamapediatrics.2017.5314; Sanderson S, Tatt ID, Higgins JP. Tools for assessing quality and susceptibility to bias in observational studies in epidemiology: a systematic review and annotated bibliography. *Int J Epidemiol*.2007;36(3):666-676; Tsang TW, Lucas BR, Carmichael Olson H, Pinto RZ, Elliott EJ. Prenatal alcohol exposure, FASD, and child behavior: a meta-analysis. *Pediatrics*. 2016;137 (3):e20152542; Hoy D, Brooks P,Woolf A, et al. Assessing risk of bias in prevalence studies: modification of an existing tool and evidence of interrater agreement.*J Clin Epidemiol*. 2012;65(9):934-939;Thomas R, Sanders S, Doust J, Beller E, Glasziou P. Prevalence of attention-deficit/hyperactivity disorder: a systematic review and meta-analysis. *Pediatrics*. 2015;135(4):e994-e1001

©2020 Laird JJ et al. *JAMA Network Open*.

eTable 3. Study Quality Scoring for Each Study Included in the Meta-analysis

	Defined Sample	Representative Sample	Cohorts in Analysis	Predictors Measured	Completion Rate	Demographic Info.	Definition Provided	Sexual Exploitation [illegible]	Publication Status	Total /9
Adjei et al,[1] 2017	1	1	1	1	1	1	1	1	1	9
Atwood et al,[2] 2012	1	1	0	1	1	1	1	1	1	8
Chang et al,[3] 2015	1	0	0	1	1	1	1	1	1	7
Chohaney,[4] 2016	1	1	1	1	0	1	1	1	1	8
Deb et al,[5] 2011	1	1	0	1	0	1	1	1	1	7
Edwards et al,[6] 2006	1	1	1	1	1	1	1	1	1	9
Fedina et al,[7] 2019	1	1	1	1		1	1	1	1	8
Fredlund et al,[8] 2013	1	1	0	1	1	1	1	1	1	8
Fredlund et al,[9] 2018	1	1	0	1	1	1	1	1	1	8
Greenbaum et al,[10] 2018	1	1	0	1	1	1	1	1	1	8
Grosso et al,[11] 2015	1	0	1	1	0	1	1	1	1	7
Ireland et al,[12] 2015	1	0	0	1	1	1	1	1	1	8
Kaestle,[13] 2012	1	1	1	1	1	1	1	1	1	9
Lavoie et al,[14] 2010	1	1	1	1	1	1	1	1	1	9
Layne et al,[15] 2014	1	1	1	1	0	1	1	1	1	8
Lung et al,[16] 2004	1	1	1	1	0	1	1	1	1	7
Martin et al,[17] 2010	1	0	1	1	0	1	1	1	1	7
Nadon et al,[18] 1998	1	0	0	1	1	1	1	1	1	7
Naramore et al,[19] 2017	1	1	1	1	1	1	1	1	1	9
O'Brien et al,[20] 2017a	1	1	1	1	1	1	1	1	1	9
O'Brien et al,[21] 2017b	1	0	1	1	1	1	1	1	1	8
Oram et al,[22] 2015	1	1	0	1	1	1	1	1	1	8
Panlilio et al,[23] 2019	1	1	0	1	1	1	1	1	1	8
Pedersen et al,[24] 2003	1	1	1	1	1	1	1	1	1	9
Reid,[25] 2011	1	0	1	1	1	1	1	1	1	8
Reid,[26] 2014	1	0	1	1	1	1	1	1	1	8
Reid et al,[27] 2014	1	0	1	1	1	1	1	1	1	8
Reid et al,[28] 2016	1	0	1	1	1	1	1	1	1	8
Saewyc et al,[29] 2010	1	1	1	1	1	1	0	1	1	8
Salisbury et al,[30] 2015	1	1	0	1	1	1	1	1	1	8
Self-Brown et al,[31] 2018	1	0	1	1	1	1	1	1	1	9
Svedin et al,[32] 2006	1	1	1	1	1	1	1	1	1	9
Swahn et al,[33] 2016	1	1	1	1	1	1	1	1	1	9
Ulloa et al,[34] 2016	1	1	1	1	1	1	1	1	1	9
Urada et al,[35] 2014	1	1	1	1	1	1	1	1	1	9
Wilson et al,[36] 2010	1	1	1	1	1	1	1	1	1	9
Yates,[37] 1991	1	1	0	1	1	1	1	1	1	8

Legend: 1 = Yes, 0 = No. Total is the summed score out of 9. The classification system utilised categorised studies into low (<2), moderate (3-5), or high quality (>6).

©2020 Laird JJ et al. *JAMA Network Open*.

eReferences

1. Adjei JK, Saewyc EM. Boys are not exempt: Sexual exploitation of adolescents in sub-Saharan Africa. *Child Abuse & Neglect*. 2017;65:14-23. doi:10.1016/j.chiabu.2017.01.001

2. Atwood KA, Kennedy SB, Shamblen S, et al. Reducing sexual risk taking behaviors among adolescents who engage in transactional sex in post-conflict Liberia. *Vulnerable Child Youth Stud*. 2012;7(1):55-65. doi:10.1080/17450128.2011.647773

3. Chang K, Lee K, Park T, Sy E, Thu Q. Using a Clinic-based Screening Tool for Primary Care Providers to Identify Commercially Sexually Exploited Children. *Journal of Applied Research on Children*. 2015;6(1):1-15.

4. Chohaney ML. Minor and adult domestic sex trafficking risk factors in Ohio. *Journal of the Society for Social Work and Research*. 2016;7(1):117-141. doi:10.1086/685108

5. Deb S, Mukherjee A, Mathews B. Aggression in Sexually Abused Trafficked Girls and Efficacy of Intervention. *Journal of Interpersonal Violence*. 2011;26(4):745-768. doi:10.1177/0886260510365875

6. Edwards JM, Iritani BJ, Hallfors DD. Prevalence and correlates of exchanging sex for drugs or money among adolescents in the United States. *Sexually Transmitted Infections*. 2006;82(5):354-358. doi:10.1136/sti.2006.020693

7. Fedina L, Williamson C, Perdue T. Risk Factors for Domestic Child Sex Trafficking in the United States. *J Interpers Violence*. 2019;34(13):2653-2673. doi:10.1177/0886260516662306

8. Fredlund C, Svensson F, Svedin CG, Priebe G, Wadsby M. Adolescents' Lifetime Experience of Selling Sex: Development Over Five Years. *J Child Sex Abuse*. 2013;22(3):312-325. doi:10.1080/10538712.2013.743950

©2020 Laird JJ et al. *JAMA Network Open*.

9. Fredlund C, Dahlström Ö, Svedin CG, Wadsby M, Jonsson LS, Priebe G. Adolescents' motives for selling sex in a welfare state – A Swedish national study. *Child Abuse & Neglect*. 2018;81:286-295. doi:10.1016/j.chiabu.2018.04.030

10. Greenbaum VJ, Livings MS, Lai BS, et al. Evaluation of a Tool to Identify Child Sex Trafficking Victims in Multiple Healthcare Settings. *Journal of Adolescent Health*. 2018;63(6):745-752. doi:10.1016/j.jadohealth.2018.06.032

11. Grosso AL, Ketende S, Dam K, et al. Structural determinants of health among women who started selling sex as minors in Burkina Faso. *J Acquir Immune Defic Syndr*. 2015;68 Suppl 2:S162-170. doi:10.1097/QAI.0000000000000447

12. Ireland CA, Alderson K, Ireland JL. Sexual Exploitation in Children: Nature, Prevalence, and Distinguishing Characteristics Reported in Young Adulthood. *Journal of Aggression, Maltreatment & Trauma*. 2015;24(6):603-622. doi:10.1080/10926771.2015.1049765

13. Kaestle CE. Selling and buying sex: A longitudinal study of risk and protective factors in adolescence. *Prev Sci*. 2012;13(3):314-322. doi:10.1007/s11121-011-0268-8

14. Lavoie F, Thibodeau C, Gagné M-H, Hébert M. Buying and selling sex in Québec adolescents: A study of risk and protective factors. *Arch Sex Behav*. 2010;39(5):1147-1160. doi:10.1007/s10508-010-9605-4

15. Layne CM, Greeson JKP, Ostrowski SA, et al. Cumulative trauma exposure and high risk behavior in adolescence: Findings from the National Child Traumatic Stress Network Core Data Set. *Psychological Trauma: Theory, Research, Practice, and Policy*. 2014;6(Suppl 1):S40-S49. doi:10.1037/a0037799

16. Lung F-W, Lin T-J, Lu Y-C, Shu B-C. Personal characteristics of adolescent prostitutes and rearing attitudes of their parents: a structural equation model. *Psychiatry Research*. 2004;125(3):285-291. doi:10.1016/j.psychres.2003.12.019

©2020 Laird JJ et al. *JAMA Network Open*.

17. Martin L, Hearst MO, Widome R. Meaningful differences: Comparison of adult women who first traded sex as a juvenile versus as an adult. *Violence Against Women*. 2010;16(11):1252-1269. doi:10.1177/1077801210386771

18. Nadon SM, Koverola C, Schludermann E H. Antecedents to prostitution: Childhood victimization. Published 1998. Accessed August 15, 2018. https://scholar-google-com.ezproxy-b.deakin.edu.au/scholar_lookup?hl=en&publication_year=1998&pages=206-221&author=S.+M.+Nadon&author=C.+Koverola&author=E.+H.+Schludermann&title=Antecedents+to+prostitution%3A+Childhood+victimization

19. Naramore R, Bright MA, Epps N, Hardt NS. Youth arrested for trading sex have the highest rates of childhood adversity: A statewide study of juvenile offenders. *Sex Abuse J Res Treat*. 2017;29(4):396-410.

20. O'Brien JE, Li W, Givens A, Leibowitz GS. Domestic minor sex trafficking among adjudicated male youth: prevalence and links to treatment. *Child Youth Serv Rev*. 2017;82:392-399. doi:10.1016/j.childyouth.2017.09.026

21. O'Brien JE, Rizo CF, White K. Domestic Minor Sex Trafficking Among Child Welfare–Involved Youth: An Exploratory Study of Correlates. *Child Maltreatment*. 2017;22(3):265-274. doi:10.1177/1077559517709995

22. Oram S, Khondoker M, Abas M, Broadbent M, Howard LM. Characteristics of trafficked adults and children with severe mental illness: A historical cohort study. *Lancet Psychiatry*. 2015;2(12):1084-1091.

23. Panlilio CC, Miyamoto S, Font SA, Schreier HMC. Assessing risk of commercial sexual exploitation among children involved in the child welfare system. *Child Abuse & Neglect*. 2019;87:88-99. doi:10.1016/j.chiabu.2018.07.021

24. Pedersen W, Hegna K. Children and adolescents who sell sex: a community study. *Social Science & Medicine*. 2003;56(1):135-147. doi:10.1016/S0277-9536(02)00015-1

25. Reid JA. An exploratory model of girl's vulnerability to commercial sexual exploitation in prostitution. *Child Maltreat*. 2011;16(2):146-157. doi:10.1177/1077559511404700

26. Reid JA. Risk and resiliency factors influencing onset and adolescence-limited commercial sexual exploitation of disadvantaged girls. *Criminal Behaviour and Mental Health*. 2014;24(5):332-344. doi:10.1002/cbm.1903

27. Reid JA, Piquero AR. Age-graded risks for commercial sexual exploitation of male and female youth. *J Interpers Violence*. 2014;29(9):1747-1777. doi:10.1177/0886260513511535

28. Reid JA, Piquero AR. Applying general strain theory to youth commercial sexual exploitation. *Crime & Delinquency*. 2016;62(3):341-367. doi:10.1177/0011128713498213

29. Saewyc EM, Edinburgh LD. Restoring healthy developmental trajectories for sexually exploited young runaway girls: Fostering protective factors and reducing risk behaviors. *J Adolesc Health*. 2010;46(2):180-188. doi:10.1016/j.jadohealth.2009.06.010

30. Salisbury EJ, Dabney JD, Russell K. Diverting victims of commercial sexual exploitation from juvenile detention: Development of the InterCSECt screening protocol. *J Interpers Violence*. 2015;30(7):1247-1276. doi:10.1177/0886260514539846

31. Self-Brown S, Culbreth R, Wilson R, Armistead L, Kasirye R, Swahn MH. Individual and Parental Risk Factors for Sexual Exploitation Among High-Risk Youth in Uganda. *J Interpers Violence*. Published online April 23, 2018:0886260518771685. doi:10.1177/0886260518771685

©2020 Laird JJ et al. *JAMA Network Open*.

32. Svedin CG, Priebe G. Selling sex in a population-based study of high school seniors in Sweden: Demographic and psychosocial correlates. *Arch Sex Behav*. 2007;36(1):21-32. doi:10.1007/s10508-006-9083-x

33. Swahn MH, Culbreth R, Salazar LF, Kasirye R, Seeley J. Prevalence of HIV and Associated Risks of Sex Work among Youth in the Slums of Kampala. *AIDS Research And Treatment*. 2016;2016:5360180-5360180. doi:10.1155/2016/5360180

34. Ulloa E, Salazar M, Monjaras L. Prevalence and Correlates of Sex Exchange Among a Nationally Representative Sample of Adolescents and Young Adults. *J Child Sex Abuse*. 2016;25(5):524-537. doi:10.1080/10538712.2016.1167802

35. Urada LA, Silverman JG, Tsai LC, Morisky DE. Underage youth trading sex in the Philippines: Trafficking and HIV risk. *AIDS Care*. 2014;26(12):1586-1591. doi:10.1080/09540121.2014.936818

36. Wilson HW, Widom CS. The role of youth problem behaviors in the path from child abuse and neglect to prostitution: A prospective examination. *J Res Adolesc*. 2010;20(1):210-236. doi:10.1111/j.1532-7795.2009.00624.x

37. Yates GL, MacKenzie RG, Pennbridge J, Swofford A. A risk profile comparison of homeless youth involved in prostitution and homeless youth not involved. *Journal of Adolescent Health*. 1991;12(7):545-548. doi:10.1016/0197-0070(91)90085-Z

©2020 Laird JJ et al. *JAMA Network Open*.
